# Extreme Quantum Advantage when Simulating Classical Systems with Long-Range Interaction

**DOI:** 10.1038/s41598-017-04928-7

**Published:** 2017-07-27

**Authors:** Cina Aghamohammadi, John R. Mahoney, James P. Crutchfield

**Affiliations:** Complexity Sciences Center and Physics Department, University of California at Davis, One Shields Avenue, Davis, CA 95616 USA

## Abstract

Classical stochastic processes can be generated by quantum simulators instead of the more standard classical ones, such as hidden Markov models. One reason for using quantum simulators has recently come to the fore: they generally require less memory than their classical counterparts. Here, we examine this quantum advantage for strongly coupled spin systems—in particular, the Dyson one-dimensional Ising spin chain with variable interaction length. We find that the advantage scales with both interaction range and temperature, growing without bound as interaction range increases. In particular, simulating Dyson’s original spin chain with the most memory-efficient classical algorithm known requires infinite memory, while a quantum simulator requires only finite memory. Thus, quantum systems can very efficiently simulate strongly coupled one-dimensional classical spin systems.

## Introduction

The idea of a quantum computer, often attributed to Feynman^1^, recognizes that while simulating quantum many-body systems is difficult, it is apparently something that the physical quantum system to be simulated itself accomplishes with ease. For this reason, it was conjectured that a “quantum computer”—one that operates on a quantum instead of classical substrate—might have a significant advantage in such a simulation. As modern computational technology approaches its quantum limits, the potential for a quantum advantage is becoming increasingly appealing. This has motivated diverse implementations of quantum hardware from trapped ions^2, 3^, cold atoms in optical lattices^4, 5^, superconducting circuits^6, 7^, photons^8, 9^ to liquid and solid-state NMR^10, 11^ and quantum dots^12^.

The phrase “quantum simulation” often refers to (as originally conceived) the simulation of a *quantum* system^13^. However, this is not the only avenue in which we find quantum advantages. For instance, there is a variety of *classical* systems that can be simulated quantally with advantage^14^ including thermal states^15^, fluid flows^16, 17^, electromagnetic fields^18^, diffusion processes^19, 20^, Burger’s equation^21^, and molecular dynamics^22^.

Quantum advantages can also be found outside of the realm of simulation. Some mathematical problems can be solved more efficiently using a quantum computer. The most well-known of these include Shor’s factorization algorithm^23^, Grover’s quantum search algorithm^24^, a quantum eigen-decomposition algorithm^25^, and a quantum linear systems algorithm^26^. For factorization, the memory required for Shor’s algorithm scales polynomially^23^ while the best classical algorithm currently known scales exponentially^27^. While neither algorithm has been proven optimal, many believe that the separation in resource scaling is real^28^.

Quantum advantages also exist in the context of stochastic process generation. Sequential generation and simultaneous generation are two important problems in this field^29^. In 1989, Crutchfield and Young^30^ introduced memory efficient classical algorithms for both of these problems. While there is a small number of known cases (processes) for which this algorithm can be surpassed^31–33^, there remains no better general classical algorithm. Our focus here is the problem of *simultaneous generation*, the potential quantum advantage therein, and the separation in classical-quantum scaling. [Quantum algorithms for sequential generation have been studied recently^34–36^.

Reference 37 provided a quantum algorithm that can generally perform simultaneous generation using less memory than the best known classical algorithms. Recently, we introduced a new quantum algorithm—the *q*-*machine*—that improved this efficiency. The latter demonstrated constructively how attention to higher-order correlations in the stochastic process can lead to an improved quantum algorithm for generation^38^. A sequel provided more detailed analysis and derived the quantum advantage of the q-machine in closed form^39^. This quantum advantage has also been verified experimentally for a simple case^40^. Just as for integer factorization, proof of optimality of a simultaneous-generation algorithm is challenging in both classical and quantum settings. However, with minor restrictions, one can show that the current quantum algorithm is almost always more efficient than the classical^38^.

While the existing results demonstrate a quantum advantage for generic processes, a significant question remains: *What is the scaling behavior of this advantage*? That is, to truly understand the nature of the advantage, it is critical to know how it depends on problem size. The strong separation in scaling between the classical and quantum integer factorization algorithms led many to expect that the separation will persist even as new algorithms are developed. We wish to demonstrate an analogous separation in scaling, thus solidifying the importance of the current quantum construction—the q-machine.

We choose as our testing ground the generation of equilibrium states for the one-dimensional Ising system with *N*-nearest neighbor interaction. Here, the coupling range *N* is our problem-size parameter. We choose a spin-spin coupling that decays as a power law in *N*. This model, having been studied in detail over four decades^41–44^, provides a physically grounded benchmark.

To understand the use of such a system in this problem context, consider a one-dimensional chain of spins (with arbitrary classical Hamiltonian) in contact with a thermal reservoir. After thermalizing, the resulting bi-infinite chain of spins (considered together at some instant *t* = *t*_0_) can be regarded as a (spatial) stochastic process. Successful generation of this stochastic process is then equivalent to generating its equilibrium states.

We quantitatively define the quantum advantage as the ratio of necessary memories for classical and quantum algorithms. Our main result is that the quantum advantage scales as *NT* ^2^/log *T* where *T* is the temperature. We also show that classically simulating Dyson’s original model requires infinite memory. In other words, exact classical simulation of the Dyson spin chain is impossible, while it is quantally possible.

### Dyson-Ising Spin Chain

We begin with a general one-dimensional classical ferromagnetic Ising spin chain^45,46^ defined by the Hamiltonian:

1 $$ {\mathcal H} =-\sum _{\langle i,j\rangle }\,J(i,j)\,{s}_{i}{s}_{j},$$

in contact with a thermal bath at temperature *T*, where *s*_*i*_, the spin at site *i*, takes on values {+1, −1}, and *J*(*i*, *j*) ≥ 0 is the spin coupling constant between sites *i* and *j*. [Throughout, *T* denotes the effective temperature *k*_*B*_ *T*]. Assuming translational symmetry, we may replace $J(i,j)$ by *J*(*k*) with $k\equiv |i-j|$. Commonly, *J*(*k*) is a positive and monotone-decreasing function. An interaction is said to be *long*-*range* if *J*(*k*) decays more slowly than exponential. In the following, we consider couplings that decay according to a power law:

2 $$J(k)=\frac{{J}_{0}}{{k}^{\delta }},$$

where *δ* > 0 and, unless otherwise noted, *J*_0_ = 1. The spin chain resulting from these assumptions is called the *Dyson* model^41^.

To approximate such an infinite-range system we consider one with finite-range interactions. For every interaction range *N*, we define the approximating Hamiltonian:

3 $${ {\mathcal H} }_{N}=-\sum _{i}\,\sum _{k=1}^{N}\,\frac{{J}_{0}}{{k}^{\delta }}{s}_{i}{s}_{i+k}.$$

[*N* is the interaction range and should not be mistaken with the size of the lattice which is infinite here]. This class of Hamiltonians can certainly be studied in its own right, not simply as an approximation. But why is the Dyson model interesting? The ferromagnetic Ising linear spin chain with finite-range interaction cannot undergo a phase transition at any positive temperature^47^. In contrast, the Dyson model has a standard second-order phase transition for a range of *δ*. Dyson analytically proved^41^ that a phase transition exists for 1 < *δ* < 2. The existence of a transition at *δ* = 2 was established much later^43^. It is also known that there exists no phase transition for *δ* > 3^42^, where it behaves as a short-range system. Finally, it was demonstrated numerically that the parameter regime 2 < *δ* ≤ 3 contains a phase transition^44^, however, this fact has resisted analytical proof. For *δ* ≤ 1, the model is considered nonphysical since the energy becomes nonextensive.

Notably, the driven quantum Dyson model has been studied experimentally of late, since it exhibits many interesting nonequilibrium phases, such as the recently introduced *discrete time crystal* ^48^. The experimental system consists of a lattice of hundreds of spin one-half particles stored in a Penning trap. Particles have been chosen to be ^9^Be^+^^49^, ^40^Ca^+^^50^ or ^171^Yb^+^^51, 52^ ions. Using a combination of static electric and magnetic fields, the Penning trap confines the ions. A general spin-spin coupling is implemented with an optical dipole force (ODF) induced by a pair of off-resonance laser beams. The ODF then produces Dyson-type interactions, where *δ* is tunable over 0 ≤ *δ* ≤ 3. Physically, *δ* = 1, 2, 3 corresponds to Coulomb-like, monopole-dipole, and dipole-dipole couplings, respectively.

For these reasons this family of Hamiltonians, derived from the Dyson spin system, offer a controlled way to investigate the consequences of nontrivial correlations.

### Simulators

The concept of a stochastic process is very general. Any physical system that exhibits stochastic dynamics in time or space may be thought of as *generating* a stochastic process. Here, we consider not time evolution, but rather the spatial “dynamic”. For example, consider a one-dimensional spin chain with arbitrary classical Hamiltonian in contact with a thermal reservoir. After thermalizing, a spin configuration at one instant of time may be thought of as having been generated left-to-right (or equivalently right-to-left). The probability distribution over these space-translation invariant configurations defines a stationary stochastic process.

We focus on *stationary*, *discrete*-*time*, *discrete*-*valued stationary stochastic processes*. Informally, such a process can be seen as a joint probability distribution ${\mathbb{P}}(\cdot )$ over the bi-infinite chain of random variables $\ldots {X}_{-1}{X}_{0}{X}_{1}\ldots$. Formally, the process denoted by ${\mathscr{P}}=\{{\mathscr{A}},{\rm{\Sigma }},{\mathbb{P}}\mathrm{(.)}\}$ is a probability space^53, 54^. Each spin random variable *X*_*i*_, $i\in {\mathbb{Z}}$, takes values in the set ${\mathscr{A}}$. For specificity, the observed symbols come from an alphabet ${\mathscr{A}}=\{\downarrow ,\uparrow \}$ of local spin states, but our results easily extend to any finite alphabet. ${\mathbb{P}}(\cdot )$ is the probability measure over the bi-infinite chain of random variables ${X}_{-\infty :\infty }=\ldots {X}_{-2}{X}_{-1}{X}_{0}{X}_{1}{X}_{2}\ldots$ and ${\rm{\Sigma }}$ is the *σ*-algebra generated by the cylinder sets in ${{\mathscr{A}}}^{\infty }$. Stationarity means that ${\mathbb{P}}(\cdot )$ is invariant under index translation. That is, ${\mathbb{P}}({X}_{i}{X}_{i+1}\cdots {X}_{i+m})={\mathbb{P}}({X}_{i+n}{X}_{i+1+n}\cdots {X}_{i+m+n})$, for all $m\in {{\mathbb{Z}}}^{+}$ and $n\in {\mathbb{Z}}$. For more information on stochastic processes generated by spin system we refer to refs. 55 and 56.

Consider a device that generates a stochastic process. We call this device a *simulator* of the process if there is no way to distinguish the process outputs from those of the simulator. Given a physical system that yields a stochastic process, a device that generates this process is then said to simulate the physical system. In some contexts, the word “simulation” implies an approximation. In contrast, we require our simulators to be exact.

How do these simulators work? Generally, we implement the algorithms by writing computer programs. Two common formalisms used as algorithms for generating stochastic processes are *Markov Chains* (MC)^57, 58^ and *Hidden Markov Models* (HMM)^53, 59, 60^. The latter can be significantly more compact in their representations (more efficient algorithms) and, for this reason, are sometimes the preferred implementation choice.

HMMs represent the generating mechanism for a given process by a tuple $\{{\mathscr{S}},{\mathscr{A}},\{{T}^{(x)}:x\in {\mathscr{A}}\}\}$, where ${\mathscr{S}}$ is a finite set of states, ${\mathscr{A}}$ is a finite alphabet (set of symbols), and $\{{T}^{(x)}:x\in {\mathscr{A}}\}$ is a set of $|{\mathscr{S}}|\times |{\mathscr{S}}|$ substochastic symbol-labeled transition matrices. The sum ${\bf{T}}={\sum }_{x\in {\mathscr{A}}}\,{T}^{(x)}$ is a stochastic matrix.

As an example, consider the Even Process^61, 62^. This process can be explained by a simple procedure. Consider a biased coin that with probability *p* generates heads and with 1−*p* generates tails. To generate the Even Process we use the algorithm:

$$\{\begin{array}{ll}Step\,A: & Flip\,the\,coin.\\ Step\,B: & If\,the\,result\,is\,heads,\,output\,0\,and\,go\,to\,Step\,A.Else\,output\,1\,and\,go\,to\,Step\,C.\\ Step\,C: & Output\,1\,and\,go\,to\,Step\,A.\end{array}$$

**Figure 1:**
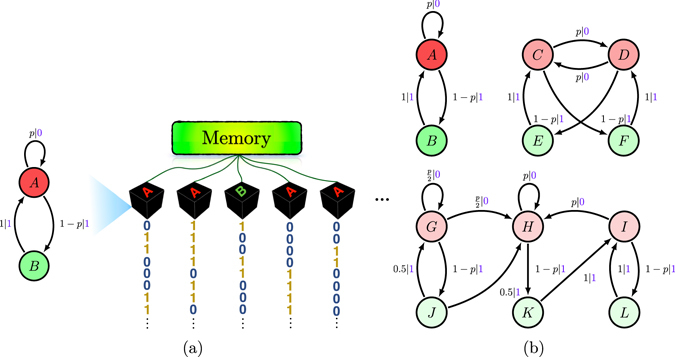
(**a**) Left: Even Process $\epsilon$-machine. Right: Schematic of simultaneous generation problem. Each black box contains an Even Process generator. They all share the same memory for tracking the individual generator states. (**b**) Alternative HMMs: Even Process generators, each with different memory costs. Top: Unifilar HMMs, since for every state and symbol there is at most one outgoing edge from that state emitting that symbol. Below: Nonunifilar HMM, since for example state *G* can go to different states *G* or *H* emitting symbol 0.

This algorithm is depicted by the HMM shown on the left of Fig. 1(a). For this HMM, ${\mathscr{S}}=\{A,B\}$, ${\mathscr{A}}=\{0,1\}$, $${T}^{\mathrm{(0)}}=(\begin{array}{cc}p & 0\\ 0 & 0\end{array})$$, and $${T}^{\mathrm{(1)}}=(\begin{array}{cc}0 & 1-p\\ 1 & 0\end{array})$$. The HMM, as an algorithm, simply tells the computer that: if we are in state *A* then, with probability *p*, output 0 and stay at state *A* and, with probability 1−*p*, output 1 and go to state *B*. If we are in state *B*, output 1 and go to state *A*.

The goal of sequential generation is to produce a very long realization of the process. For this, we use one computer with a code that runs the algorithm. At each step, the computer must memorize the current HMM state. Since the HMM has 2 states, we require 1 bit of memory for this process, independent of its bias *p*.

Here, though, we are interested in simultaneous generation where the goal is to generate *M* realizations of a process simultaneously, each of which is statistically independent of the others. The net result is *M* computers each with the above code, as on the right side of Fig. 1(a). Similar to the sequential problem, each computer must memorize the current state of its HMM. If each computer uses its own memory, each needs 1 bit of memory as before. The total memory is then *M* bits.

However, we can reduce the amount of memory required by using one large shared memory among the computers. Figure 1(a) emphasizes this schematically. In this way, according to Shannon’s coding theorem^63^, we can encode the HMM states to reduce the amount of memory to $M\,{\rm{H}}({\mathscr{S}})\le M$ bits, where ${\rm{H}}({\mathscr{S}})=$ $-{\rm{\Pr }}(A)\,{\mathrm{log}}_{2}\,{\rm{\Pr }}(A)-{\rm{\Pr }}(B)\,{\mathrm{log}}_{2}\,{\rm{\Pr }}(B)$. The memory per instance is then just ${\rm{H}}({\mathscr{S}})$.

Every process has an infinite number of alternative HMMs that generate it. For example, Fig. 1(b) shows three HMMs that each generate the Even Process, each with different ${\rm{H}}({\mathscr{S}})$ and as a result different memory costs. Now, an important question when considering all possible generators is, which HMM needs the minimum memory or, equivalently, minimum ${\rm{H}}({\mathscr{S}})$?

#### *∈*-Machine

A *unifilar* HMM is one in which each row of each substochastic matrix has at most one nonzero element. Informally, this means the current state and next symbol uniquely determine the next state. Many statistical and informational quantities can be calculated in closed form from a process’s unifilar HMM; see ref. 64 and discussion therein. For example, in Fig. 1(b) the top two HMMs are unifilar and the bottom one is nonunifilar.

For a given process, finding the optimal HMM for simultaneous generation—an HMM with minimum state-entropy ${\rm{H}}({\mathscr{S}})$—in the space of all HMMs is still an open question. Restricting to the space of unifilar HMMs, though, the optimal HMM can be found. It is the $\epsilon$-*machine* ^65^, first introduced in ref. 30. $\epsilon$-Machine states ${\bf{S}}$ are called *causal states*. Due to the $\epsilon$-machine’s unifilarity property, every generated past maps to a unique causal state. A process’ *statistical complexity C*_*μ*_^65^ is the the Shannon entropy of the $\epsilon$-machine’s stationary state distribution: ${C}_{\mu }={\rm{H}}({\mathscr{S}})=-{\sum }_{\sigma \in {\mathscr{S}}}\,{\rm{\Pr }}(\sigma )\,{\mathrm{log}}_{2}\,{\rm{\Pr }}(\sigma )$. This is the required memory for simultaneous generation.

Attempts have been made to find smaller models among *nonunifilar* HMMs^31^. As of now, though, only a handful of examples exist^31–33, 66^. Practically speaking, the $\epsilon$-machine is the most memory-efficient algorithm for generating stochastic processes. Its memory *C*_*μ*_ has been determined for a wide range of physical systems^67–73^. Helpfully, it and companion informational measures are directly calculable from the $\epsilon$-machine, many in closed-form^64^.

We denote the process generated by the physical system with Hamiltonian Eq. (3) wi﻿th *J*_0_ = 1 at temperature *T* by ${\mathscr{P}}(N,T)$. How do we construct the $\epsilon$-machine that simulates the process ${\mathscr{P}}(N,T)$? First, we must define a process’ *Markov order* ^58^: the minimum history length *R* required by any simulator to correctly continue a configuration. Specifically, *R* is the smallest integer such that:

4 $${\mathbb{P}}({X}_{t}|\ldots ,{X}_{t-2},{X}_{t-1})={\mathbb{P}}({X}_{t}|{X}_{t-R},\ldots ,{X}_{t-2},{X}_{t-1}).$$

[More precisely, an ensemble of simulators must yield an ensemble of configurations that agree (conditioned on that past) with the process’ configuration distribution].

**Figure 2:**
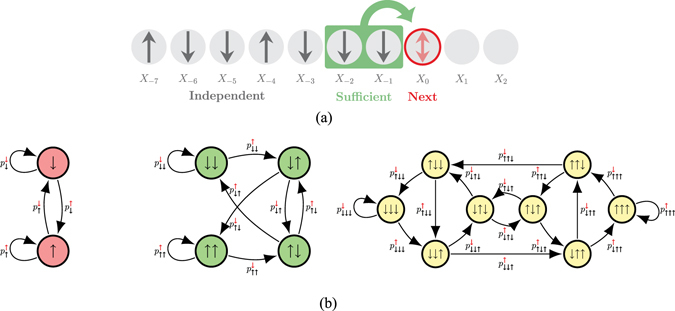
(**a**) A Markov order-*N* process generates a spin configuration from left-to-right. Markov order *N* = 2 shown. The values of an isolated spin *X*_0_, say, is undetermined. To make this (stochastic) choice consistent with the overall process and the particular instantiation on the left, it is sufficient to consider only the previous *N* (2) spins (highlighted in green). (**b**) $\epsilon$-Machine generators of 1D-configuration stochastic processes in Dyson-Ising systems of increasing correlational complexity (*N* = 1, 2, 3): ${\mathscr{P}}(1,T)$ (left), ${\mathscr{P}}(2,T)$ (middle) and ${\mathscr{P}}(3,T)$ (right).

Reference [56, Eqs (84)–(91)] showed that for any finite and nonzero temperature *T*, ${\mathscr{P}}(N,T)$ has Markov order *N*. One concludes that sufficient information for generation is contained in the configuration of the *N* previously generated spins. (Figure 2(a) shows this fact schematically for *N* = 2). More importantly, the $\epsilon$-machine that simulates ${\mathscr{P}}(N,T)$ has 2^*N*^ causal states and those states are in one-to-one correspondence with the set of length-*N* spin configurations.

Second, another key process characteristic is its *cryptic order* ^74, 75^: the smallest integer *K* such that ${\rm{H}}[{{\mathscr{S}}}_{K}|{X}_{0}{X}_{1}\ldots ]=0$, where H[*W*|*Z*] is the conditional entropy^63^ and ${{\mathscr{S}}}_{K}$ is the random variable for *K* ^*th*^ state of the $\epsilon$-machine after generating symbols ${X}_{0},{X}_{1},\ldots ,{X}_{K-1}$. Using the fact that the $\epsilon$-machine’s states are in one-to-one correspondence with the set of length-*N* spin configurations^56^, it is easy to see that ${\mathscr{P}}(N,T)$’s cryptic order *K* = *N*, the Markov order. We will use this fact in the quantum algorithm construction to follow.

Figure 2(b) shows the $\epsilon$-machine of the processes ${\mathscr{P}}(N,T)$ for *N* = 1, 2, and 3. Let’s explain. First, consider the spin process ${\mathscr{P}}\mathrm{(1,}\,T)$ that, as we pointed out, is a Markov-order *R* = 1 process. This means that to generate the process the simulator only need remember the last spin generated. In turn, this means the $\epsilon$-machine (Fig. 2(b) left) has two states, ↑ and ↓. If the last observed spin is ↑, the current state is ↑ and if it is ↓, the current state is ↓. We denote the probability of generating a  spin given a previous generated ↑ spin by . The probability of an  spin following a ↑ spin is the complement.

Second, consider the process ${\mathscr{P}}\mathrm{(2,}\,T)$ with Markov-order *R* = 2 and so longer-range interactions. Sufficient information for generation is contained in the configuration of the two previously generated spins. Thus, the $\epsilon$-machine (Fig. 2(b) middle) has four states that we naturally label ↑↑, ↑↓, ↓↑, and ↓↓. If the last observed spin pair ${x}_{-1}{x}_{0}$ is ↑↓, the current state is ↑↓. Given this state, the next spin will be  with probability  and  with probability . Note that this scheme implies that each state has exactly two outgoing transitions. That is, not all state-to-state transitions are allowed in the $\epsilon$-machine.

Having identified the state space, we complete the $\epsilon$-machine construction by determining the $\epsilon$-machine transition probabilities ${\{{T}^{(x)}\}}_{x\in {\mathscr{A}}}$. To do this, we first compute the transfer matrix *V* for the Ising *N*-nearest neighbors with the Hamiltonian in Eq. (3) at temperature *T* and then extract conditional probabilities, following ref. 56. (See the Method section following for details.) The minimum memory for simultaneous generation or, as it is called, the statistical complexity *C*_*μ*_(*N*, *T*) of process ${\mathscr{P}}(N,T)$ follows straightforwardly from the process’ $\epsilon$-machine.

#### q-Machine

By studying a specific process (similar to the $\epsilon$-machine in left of Fig. 2(b)), ref. 37 recently demonstrated that quantum mechanics can generate stochastic processes using less memory than *C*_*μ*_. This motivates a search for more efficient quantum simulators of processes with richer correlational structure.

A process’ quantum simulator is a pair $\{f, {\mathcal M} \}$, where $f:{{\mathscr{A}}}^{\infty }\to {\rm{\Omega }}$ is a function from the set ${{\mathscr{A}}}^{\infty }$ of past sequences to a set of quantum states Ω and ${\mathcal M}$ is some measurement process. Given a particular past ${x}_{-\infty \mathrm{:0}}$, applying the measurement ${\mathcal M}$ to the quantum state $f({x}_{-\infty \mathrm{:0}})$ leads to a correct probability distribution over futures ${\mathbb{P}}({x}_{0:n}|{x}_{-\infty :0})$. If *f*(·) is a deterministic function, the simulator is called unifilar and if *f* is a probabilistic function, the simulator is called nonunifilar. After generating ${x}_{\mathrm{0:}n}$, the new past is ${x}_{-\infty :n}$ and *f* can be used to map it to the new quantum state $f({x}_{-\infty :n})$. By repeating the same measurement and mapping procedure, we generate a realization of the process. One can also define the quantum simulator in a way that ${\mathcal M}$ automatically maps $f({x}_{-\infty \mathrm{:0}})$ to the correct quantum state $f({x}_{-\infty :n})$ and generates the correct probability distribution over ${x}_{\mathrm{0:}n}$ ^76^.

Reference 38 introduced a class of unifilar simulators, called *q*-*machines*, that can generate arbitrary processes. As in the classical setting, nonunifilar quantum simulators are much less well understood^34, 35, 66^. The q-machine construction depends on an encoding length *L*, each with its own quantum cost *C*_*q*_(*L*). Each of these simulators simulates the same process correctly. It is known that the cost *C*_*q*_(*L*) is constant for *L* at or greater than  the process’ cryptic order^75^. Based on numerical evidence, it is conjectured that this is also the minimal *C*_*q*_ value. Thus, we restrict ourselves to this choice (*L* = *K*) of encoding length and refer simply to the q-machine and its cost *C*_*q*_.

The q-machine’s quantum memory *C*_*q*_ is upper-bounded by *C*_*μ*_, with equality only for the special class of zero-cryptic-order processes^75^. And so, *C*_*μ*_/*C*_*q*_ gives us our quantitative measure of *quantum advantage*.

Reference 39 recently introduced efficient methods for calculating *C*_*q*_ using spectral decomposition. Those results strongly suggest that the q-machine is the most memory-efficient among all unifilar quantum simulators, but as yet there is no proof. The quantum advantage *C*_*μ*_/*C*_*q*_ has been investigated both analytically^38, 39, 76–78^ and experimentally^40^.

A process’ q-machine is straightforward to construct from its $\epsilon$-machine. First, since the $\epsilon$-machine is unifilar, every generated past realization maps to a unique causal state. Second, every causal state *σ*_*i*_ maps to a pure quantum state $|{\eta }_{i}\rangle$. Thus, we can map every generated past to a unique quantum state. Each signal state $|{\eta }_{i}\rangle$ encodes the set of length-*K* (cryptic order) sequences that may follow *σ*_*i*_, as well as each corresponding conditional probability:

5 $$|{\eta }_{i}\rangle \equiv \sum _{w\in {{\mathscr{A}}}^{K}}\,\sum _{{\sigma }_{j}\in {\mathscr{S}}}\,\sqrt{{\mathbb{P}}(w,{\sigma }_{j}|{\sigma }_{i})}\,|w\rangle |{\sigma }_{j}\rangle ,$$

where *w* denotes a length-*K* sequence and ${\mathbb{P}}(w,{\sigma }_{j}|{\sigma }_{i})={\mathbb{P}}({X}_{0}\cdots {X}_{K-1}=w,{{\mathscr{S}}}_{K-1}={\sigma }_{j}|{{\mathscr{S}}}_{0}={\sigma }_{i})$. The resulting Hilbert space is the product ${ {\mathcal H} }_{w}\otimes { {\mathcal H} }_{\sigma }$. Factor space ${ {\mathcal H} }_{\sigma }$ is of size $|{\mathscr{S}}|$, the number of classical causal states, with basis elements |*σ*_*i*_〉. Factor space ${ {\mathcal H} }_{w}$ is of size ${|{\mathscr{A}}|}^{K}$, the number of length-*K* sequences, with basis elements $|w\rangle =|{x}_{0}\rangle \cdots |{x}_{K-1}\rangle$. Performing a projective measurement in the $|w\rangle |\sigma \rangle$ basis results in a correct probability distribution. After generation of a particular realization by measurement, the next corresponding quantum state can be indicated uniquely. This means we can repeat the measurement process and continue generating the process.

Now, let us return to simultaneous generation where the goal is to generate *M* process realizations simultaneously where each is statistically independent of the others. As before, we have *M* q-machines as in Fig. 1. Also similar to the classical setting, we can reduce the amount of required memory by having the q-machines use a single shared memory. According to the Schumacher noiseless  quantum coding theorem, we can encode the HMM states to reduce the amount of memory to *MS*(*ρ*) qubits where *S*(·) is von Neumann entropy and *ρ* is the density matrix defined by:

6 $$\rho =\sum _{i}\,{\pi }_{i}|{\eta }_{i}\rangle \langle {\eta }_{i}|.$$

As a result, each q-machine needs *C*_*q*_ = *S*(*ρ*) qubits of memory for simultaneous generation.

### Analysis

**Figure 3:**
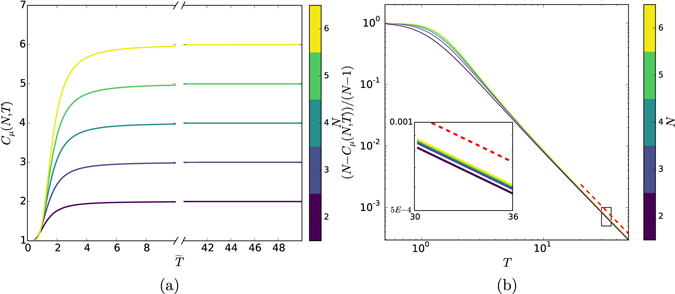
(**a**) Classical memory *C*_*μ*_(*N*, *T*) required for simulating process ${\mathscr{P}}(N,T)$ for interaction ranges $N=1,\ldots ,6$, a range of temperatures $T=1,\ldots ,50$, and *δ* = 2. Note *C*_*μ*_(·) is an increasing function of *N* and *T*. (**b**) Rescaling the classical memory requirement *C*_*μ*_(*N*, *T*) to (*N* − *C*_*μ*_)/(*N* − 1) shows a tight data collapse, which is especially strong at high temperatures (*T* > 2). The asymptotic behavior is a power law with scaling exponent *γ* = 2. The inset zooms in to show *C*_*μ*_’s convergence with increasing *N*. While the figure shows the case *δ* = 2, the slope *γ* at high *T* is independent of *δ*.

**Figure 4:**
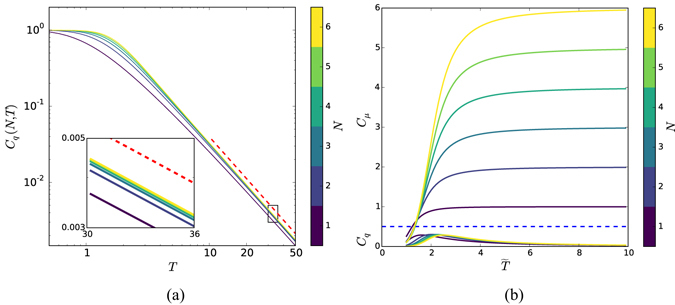
(**a﻿﻿**) C_*q*_(·) is an increasing function of *N*, but a decreasing function of *T* and bounded by 1 qubit, independent of *N* and *T*. Quantum memory *C*_*q*_(*N*, *T*), similar to *C*_*μ*_(*N*, *T*), shows a data collapse in *N* that is especially tight at high temperature (*T* > 2). The asymptotic behavior is a power law with numerically estimated scaling exponent *α* = 2. (Red dashed line.) The lower inset zooms to highlight convergence with increasing *N*. Though the curves are for the case with *δ* = 2, the slope *α* at high *T* is independent of *T*. (**b**) Magnetic field effects on classical *C*_*μ*_(*N*, *T*) and quantum *C*_*q*_(*N*, *T*) memory requirements for simulating the processes generated by Hamiltonian ${\widehat{ {\mathcal H} }}_{N}$ for *N* = 1, …, 6 over a range of temperatures *T* = 1, …, 10 at *B* = 0.3. *C*_*q*_(*N*, *T*) curves are those under the dashed blue line.

We begin by considering the case where spin couplings decay with exponent *δ* = 2. Figure 3(a) displays *C*_*μ*_(*N*, *T*) and Fig. 4(a) displays *C*_*q*_(*N*, *T*)—the *C*_*μ*_ and *C*_*q*_ of processes ${\mathscr{P}}(N,T)$—versus *T* for interaction ranges *N* = 1, …, 6. The most striking feature is that the classical and quantum memory requirements exhibit qualitatively different behaviors.

The classical memory increases with *T*, saturating at *C*_*μ*_ = *N*, since all transitions become equally likely at high temperature. As a result there are 2^*N*^ equally probable causal states and this means one needs *N* bits of memory to store the system’s current state. For example, in the nearest-neighbor Ising model (process ${\mathscr{P}}\mathrm{(1,}\,T)$) high temperature makes spin-↑ and spin-↓, and thus the corresponding states, equally likely. [At *T* = ∞ these processes have only a single causal state and thus *C*_*μ*_ = 0. This is a well-known discontinuity that derives from the sudden predictive-equivalence of all of the causal states there.]

Also, in the low-temperature limit, this system is known to yield one of only two equally likely configurations—all spin-↑ or all spin-↓. In other words, at low temperature  and  converge to zero, while  and  converge to one. [It should be pointed out that at any finite temperature  and  are nonzero and, therefore, the $\epsilon$-machine states remains strongly-connected.] This is reflected in the convergence of all curves at *C*_*μ*_ = 1 bit. Equivalently, this means one needs only a single bit of memory to store the current state.

We can similarly understand the qualitative behavior of *C*_*q*_(*N*, *T*) for a fixed *N*. As temperature increases, all length-*N* signal states become equivalent. This is the same as saying that independent of the previous spins all the next length-*N* spin configurations become equally likely. As a consequence, the signal states approach one another and, thus, *C*_*q*_(*N*, *T*) converges to zero.

In the low temperature limit, the two *N*-↑ and *N*-↓ configurations are distinguished by the high likelihood of neighboring spins being of like type. This leads to a von Neumann entropy (*C*_*q*_) of *S*(*ρ*) = 1 qubit.

Figure 3(a) reveals strong similarities in the form of *C*_*μ*_(*T*) at different *N*. A simple linear scaling leads to a substantial data collapse, shown in Fig. 3(b). The scaled curves (*N* − *C*_*μ*_)/(*N* − 1) exhibit power law behavior in *T* for *T* > 2. Increasing the temperature to *T* = 300 (beyond the scale in Fig. 3(b)) numerical estimates from simulations indicate that this scaling is given by $\gamma \simeq 2.000$. The scaling determines how the classical memory saturates at high temperature.

This behavior is generic for different coupling decay values *δ* > 1 and, more to the point, the scaling is independent of *δ*. We do not consider *δ* < 1, where the system energy becomes nonextensive.

Now, we can analyze the decrease in *C*_*q*_ with temperature. Figure 4(a) shows that *C*_*q*_ is also a power law in *T*. By measuring this scaling exponent in the same way as above, we determined that $\alpha \simeq 2.000$. Furthermore, we find analytically that for high *T*:

7 $${C}_{q}(N,T)\propto \frac{{\mathrm{log}}_{2}\,(T)}{{T}^{2}}.$$

To see this, first consider nearest-neighbor coupling *N* = 1. Due to symmetry we have $p\equiv {\rm{\Pr }}(\uparrow |\uparrow )={\rm{\Pr }}(\downarrow |\downarrow )\,=\,$ $F/D$, where *F* = exp(*β*) and $D=\exp (\beta )+\sqrt{\exp (-2\beta )}$ with *β* = 1/*T*. At high temperature *β* is small and we have *D* = 2 + *β* ^2^ and *F* = 1 + *β* + *β* ^2^. Again, by symmetry we have ${\pi }_{1}={\pi }_{2}=1/2$ and, therefore, the density matrix in Eq. (6) is:

8 $$\rho =(\begin{array}{cc}1/2 & \sqrt{p\mathrm{(1}-p)}\\ \sqrt{p\mathrm{(1}-p)} & 1/2\end{array}),$$

which has two eigenvalues: *β* ^2^/4 and 1 − *β* ^2^/4. As a consequence *C*_*q*_, being *ρ*’s von Neumann entropy, is:

9 $${C}_{q}=S(\rho )\simeq -(\frac{{\beta }^{2}}{4}\,{\mathrm{log}}_{2}\frac{{\beta }^{2}}{4}+(1-\frac{{\beta }^{2}}{4})\,{\mathrm{log}}_{2}\,(1-\frac{{\beta }^{2}}{4}))\simeq \frac{{\mathrm{log}}_{2}\,(T)}{2{T}^{2}}.$$

Examining the numerator, for any *r* > 0 we have ${\mathrm{log}}_{2}(T) < {T}^{r}$. So, for large *T*:

10 $$\frac{1}{{T}^{2}} < \frac{{\mathrm{log}}_{2}\,(T)}{{T}^{2}} < \frac{1}{{T}^{2-r}},$$

for all *r* > 0. This explains the fat tails of *C*_*q*_ for large *T* and establishes that for *N* = 1 the scaling exponent is *α* = 2.

Increasing the temperature the link between spins weakens. At high temperature the only important neighbor is the nearest. As a consequence, the high temperature behavior is similar to the case of *N* = 1 and, in addition, it is independent of *N*. This verifies and adds detail to our numerical estimate.

This behavior is generic for different coupling decay values *δ* > 1 and, moreover, the scaling exponent *α* is independent of *δ*. Notably, in this case no rescaling is required. The exponent directly captures the extreme compactness of high-temperature quantum simulations.

Taking these results together, we can now appreciate the substantial relative advantage of quantum versus classical simulators.

Define the *quantum advantage η* as the ratio of the minimum required memory for the classical simulation to the minimum required memory for the quantum simulation:

11 $$\eta (N,T)\equiv {C}_{\mu }(N,T)/{C}_{q}(N,T).$$

For fixed temperature $T\,\gtrapprox \,2$, *C*_*μ*_ (*N*, *T*) is approximately linear in *N* and for a fixed *N* is approximately independent of *T*. As a consequence, the asymptotic quantum advantage is:

12 $$\eta (N,T)\propto N\frac{{T}^{2}}{{\mathrm{log}}_{2}\,(T)},$$

which scales faster than any *T* ^*r*^ for *r* < 2. Thus, the answer to our motivating question is that the quantum advantage does, in fact, display scaling: it increases with interaction range *N* and also increases strongly with temperature *T*.

Up to this point we focused on finite interaction-range systems, interpreting the chosen models as a family of approximations to the Dyson model. Consider, though, Dyson’s original spin chain^41^ which has infinite-range interactions. In this case, the classical memory cost of simulation diverges: ${\mathrm{lim}}_{N\to \infty }{C}_{\mu }(N,T)\to \infty$. That is, it is impossible to simulate the Dyson model classically. In contrast, the quantum memory cost is finite—${\mathrm{lim}}_{N\to \infty }{C}_{q}(N,T) < 1$ qubit—and so it can be simulated quantally. There is perhaps no clearer statement of quantum advantage.

Naturally, one might ask how our results are modified by the presence of an external magnetic field. Consider the one-dimensional ferromagnetic Ising spin chain with Hamiltonian:

13 $${\widehat{ {\mathcal H} }}_{N}=-\sum _{i}\,\sum _{k=1}^{N}\,\frac{{J}_{0}}{{k}^{\delta }}{s}_{i}{s}_{i+k}-\sum _{i}\,B{s}_{i}.$$

Figure 4(b) shows that, due to symmetry breaking at low temperature, both *C*_*q*_(*N*, *T*) and *C*_*μ*_(*N*, *T*) converge to zero. (All spins at low temperature align with magnetic field and, as a consequence, no memory is needed.) The high temperature behaviors for both memory costs are the same as before, though, and the quantum advantage remains the same.

## Discussion

It is notoriously hard to find quantum advantage and even harder to prove^79^. We found such an advantage in the realm of stochastic process simulation. Concretely, we analyzed the *N*-nearest neighbor Ising spin system and demonstrated that its quantum advantage displays a generic scaling behavior—quadratic in temperature and linear in interaction range. What does this mean? The most striking conclusion is that a strongly interacting classical system can be simulated with unbounded quantum advantage. One stark contrast is that it is impossible to classically simulate Dyson’s original spin chain while quantum simulators can do so and with finite memory cost.

How broadly might we expect to see this quantum advantage? Or, is it merely a feature of strongly coupled spin systems? Define a *universal* spin model as one that can simulate any other spin model. That is, by using the low-energy sector of such universal models, the physics of every classical spin model can be reproduced. Recently, ref. 80 showed that the 2D Ising model with external fields is universal in this sense. This suggests that the quantum advantage described here may not be limited to the particular spin system we consider, but might also be universal. As a result, one should expect to see the quantum advantage for other physical systems.

The Ising model has lent great insight to condensed matter physics, however it is a classical model. Given that we are examining the difference between classical and quantum simulators, it is natural to wonder about this difference in the context of a truly quantum Hamiltonian. Is the quantum advantage amplified? Are there systems for which we find no quantum advantage? And, is this their defining characteristic?

Here, we studied the cost of exact simulation of stochastic processes. Both classical and quantum costs, though, can be very different when approximation is allowed. For example, at high (but finite) temperature, we can approximate the process ${\mathscr{P}}(N,T)$ as independent, identically distribution (IID). One does not require any classical or quantum memory to generate an IID process and, as a result, there would be no quantum advantage. Apparently, the difference between required classical memory for exact simulation and approximate simulation can be quite large. In contrast, the price we pay to go from approximate to exact quantum simulation is relatively small.

## Methods

We show how to construct the $\epsilon$-machine simulator of the process ${\mathscr{P}}(N,T)$, following ref. 81. Consider a block of spins of length 2*N*, divided equally into two blocks. We denote spins in the left (L) and right (R) halves by: ${s}_{i}^{L}$ and ${s}_{i}^{R}$ for $i=1,\ldots ,N$, respectively. We map the left and right block configurations each to an integer ${\eta }_{\ast }$ by:

14 $${\eta }_{\ast }=\sum _{i=1}^{N}\,(\frac{{s}_{i}^{\ast }+1}{2})\,{2}^{i-1},$$

where * can be either *L* or *R*. For each block we can have 2^*N*^ different configurations. Consequently, the label ${\eta }_{\ast }$ varies between 0 and 2^*N*^ − 1. The internal energy of a given block with configuration ${\eta }_{\ast }$ is given by:

15 $${X}_{{\eta }_{\ast }}=-B\sum _{i=1}^{N}\,{s}_{i}^{\ast }-\sum _{i=1}^{N-1}\,\sum _{k=1}^{N-i}\,{J}_{i}{s}_{k}^{\ast }{s}_{k+i}^{\ast },$$

and the interaction energy between two blocks is:

16 $${Y}_{{\eta }_{L},{\eta }_{R}}=-\sum _{i=1}^{N}\,\sum _{k=1}^{i}\,{J}_{i}{s}_{N-k+1}^{L}{s}_{k}^{R}.$$

With these we construct the transfer matrix:

17 $${V}_{{\eta }_{L},{\eta }_{R}}={e}^{-\mathrm{(1}/2{X}_{{\eta }_{L}}+{Y}_{{\eta }_{L},{\eta }_{R}}+1/2{X}_{{\eta }_{R}})/T}.$$

The right eigenvector of *V* corresponding to the largest eigenvalue is denoted by *u*. Reference 56 shows that the $\epsilon$-machine labeled-transition matrices can be written as:

18 $${T}_{{\eta }_{0},{\eta }_{1}}^{(x)}=(\begin{array}{ll}\frac{1}{\lambda }{V}_{{\eta }_{0},{\eta }_{1}}\frac{{u}_{{\eta }_{1}}}{{u}_{{\eta }_{0}}}, & {\eta }_{1}=(\lfloor \frac{{\eta }_{0}}{2}\rfloor +x({2}^{N-1}))\\ \mathrm{0,} & {\rm{otherwise}}\end{array},$$

where *x* ∈ {0, 1}, 0 for spin down and 1 for spin up. Then, the $\epsilon$-machine simulator of ${\mathscr{P}}(N,T)$ is $\{{\mathscr{S}},{\mathscr{A}},{\{{T}^{(x)}\}}_{x\in {\mathscr{A}}}\}$, where ${\mathscr{A}}=\{0,1\}$ and ${\mathscr{S}}=\{i:0\le i\le {2}^{N}-1\}$.
